# Genomic analysis of *Bacillus cereus* NWUAB01 and its heavy metal removal from polluted soil

**DOI:** 10.1038/s41598-020-75170-x

**Published:** 2020-11-12

**Authors:** Ayansina Segun Ayangbenro, Olubukola Oluranti Babalola

**Affiliations:** Food Security and Safety, Faculty of Natural and Agricultural Sciences, North-West University, Private Bag X2046, Mmabatho, 2735 South Africa

**Keywords:** Microbiology, Environmental sciences

## Abstract

Microorganisms that display unique biotechnological characteristics are usually selected for industrial applications. *Bacillus cereus* NWUAB01 was isolated from a mining soil and its heavy metal resistance was determined on Luria–Bertani agar. The biosurfactant production was determined by screening methods such as drop collapse, emulsification and surface tension measurement. The biosurfactant produced was evaluated for metal removal (100 mg/L of each metal) from contaminated soil. The genome of the organism was sequenced using Illumina Miseq platform. Strain NWUAB01 tolerated 200 mg/L of Cd and Cr, and was also tolerant to 1000 mg/L of Pb. The biosurfactant was characterised as a lipopeptide with a metal-complexing property. The biosurfactant had a surface tension of 39.5 mN/m with metal removal efficiency of 69%, 54% and 43% for Pb, Cd and Cr respectively. The genome revealed genes responsible for metal transport/resistance and biosynthetic gene clusters involved in the synthesis of various secondary metabolites. Putative genes for transport/resistance to cadmium, chromium, copper, arsenic, lead and zinc were present in the genome. Genes responsible for biopolymer synthesis were also present in the genome. This study highlights biosurfactant production and heavy metal removal of strain NWUAB01 that can be harnessed for biotechnological applications.

## Introduction

Industrialisation and mining activities have continued to put an increasing burden on the environment as a result of metal pollution^1^. The unrestrained release of metals into the environment from these activities poses a threat to the ecosystem and health of living organisms. Mining industries, fertilizer and pesticide production release cadmium into the environment^2^. Mining, electroplating, paints and pigments, batteries, tanning and textile industries release chromium and lead into the environment^2–4^. Heavy metals have been known to cause various diseases and ailments in humans, for example, cadmium causes bone disease, headache, hypertension, kidney diseases, lung and prostate cancer^2,3,5^. Chromium causes chronic bronchitis, skin irritation, liver diseases, renal failure and lung cancer^4,6^ while lead causes chronic nephropathy insomnia, learning disorder, renal damage, reduced fertility and is a risk factor for Alzheimer’s disease^2,5,7^.

Conventional methods of heavy metal removal involve treatment with chelating agents, organic and inorganic acids, reverse osmosis, surfactants and water. However, these techniques are often expensive and ineffective for low metal concentration removal^1,5^. Other challenges often encountered with the use of these conventional techniques include non-specificity of these methods, space requirements, impractical nature of some techniques and high energy demand^1,8^. Thus, there is the need for bioremediation using microorganisms with potential for remediation of polluted environments and production of eco-friendly secondary metabolites^9^.

Bioremediation of heavy metals offers an alternative and effective means of decontaminating metal-polluted environments. Heavy metal remediation of contaminated environment mediated by microorganisms is efficient and cost effective^8^. Microorganisms have developed various mechanisms for detoxifying heavy metals. These mechanisms include biosorption, biotransformation, bioaccumulation, and biomineralisation^10^. These organisms also secrete a range of metal-sequestering polymers that are employed in metal uptake^11,12^. These biopolymers also trap and absorb metal sulphides and oxides^12^.

The use of microbial biopolymers to enhance metal removal effectiveness is emerging as a promising technique. Similarly, these polymers can survive different pH and temperature range^13^. Their metal-binding capability depends on the producing organism, functional groups on the biopolymer, metal affinity and specificity, temperature and pH^13–15^. They are eco-friendly, versatile and economic compared to chemical polymers.

One of the numerous polymers of microbial origin is biosurfactant, with various applications in detergents, cosmetics, medicine, food industries, petroleum and bioremediation^16^. There have been various reports in literature on the metal-complexing abilities of biosurfactants in removing heavy metals from polluted soil and wastewaters^15,17–19^. They solubilise metal ions through increased wettability and reduced surface tension, thereby bringing metal ions out of the soil matrix^17^. Biosurfactants of microbial origin are good metal-complexing agents due to their stability, degradability, low toxicity and environmental compatibility^20^. They form stable complexes with metal ions as a result of electrostatic interaction between charged polymers^18^. With advances in genome sequencing, different microbial products have gained increasing attention through elucidation and prediction of biosynthetic genes.

In this study, we present the genome sequence of *Bacillus cereus* NWUAB01 and its underlying genetic information associated with pollutants’ degradation and resistance. In addition, based on the nature of biosurfactants and their several applications in reclamation of polluted sites, their application in the removal of cadmium, chromium and lead, which have been listed among toxic elements within the first twenty pollutants priority list that are of significance to public health^21^ was also investigated.

## Results

### Strain identification, characteristics and tolerance to heavy metals

**Figure 1:**
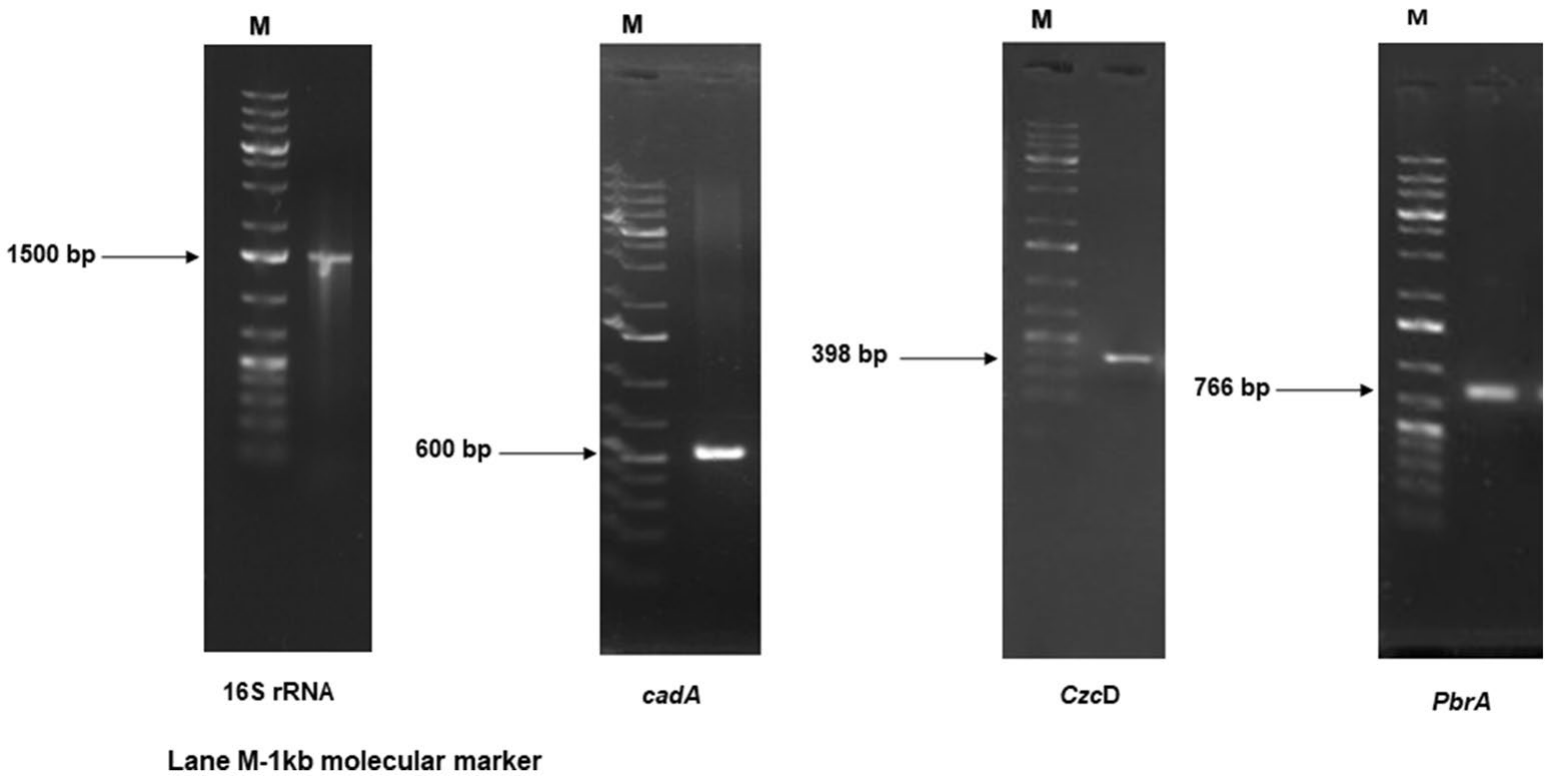
The 16S rRNA and heavy metal resistant genes amplification of deoxyribonucleic acid (DNA) sequence of strain NWUAB01.

Ninety-eight heavy metal resistant bacterial isolates were isolated from the soil samples collected and one of the isolates was identified as *B. cereus* NWUAB01. Gram staining showed that the organism is Gram-positive and has a rod shape. The biochemical profile of the isolate revealed that it can ferment glucose, fructose, sucrose, and starch. It can use citrate as a carbon source, and was catalase and nitrate positive. The organism is indole and Voges–Proskaeur negative and does not ferment mannose, sorbitol, melibiose, maltose and lactose. The amplification of the 16S ribosomal ribonucleic acid (rRNA) gene of strain NWUAB01 yielded the predicted 1500 bp amplicon (Fig. 1). The amplicon sequence was compared with the 16S rRNA gene sequences in the National Centre for Biotechnology Information (NCBI) database and it showed that strain NWUAB01 had 100% similarity with *Bacillus cereus* strain BS16 (MH021873), *B. wiedmanni* strain F23 (MF681995), *B. thuringiensis* strain FDB-6 (MH260380), *Bacillus* sp strain SP9 (MH191109) and 99% similarity with *B. proteolticus* strain SPB3 (MG280785) with E-value of 0.00.

**Figure 2:**
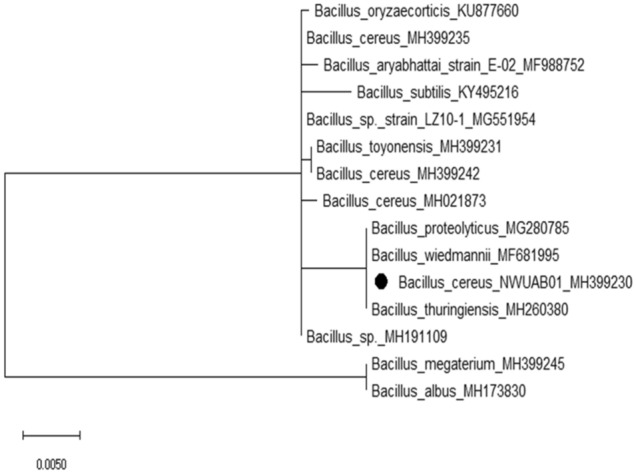
Phylogenetic tree using maximum likelihood method of strain NWUAB01 based on 16S rRNA gene sequence. The tree was generated using MEGAX software version 10.0.4 (Kumar et al.^70^) (https://www.megasoftware.net/resources).

The evolutionary relationship of strain NWUAB01 was deduced using the maximum likelihood method based on the Hasegawa–Kishino–Yano model^22^. The phylogenetic relationship of strain NWUAB01 is presented in Fig. 2, which shows the relationship of the organism with closely related strains from the GenBank. The initial tree for the heuristic search was obtained automatically by applying the neighbour-join method. The tree is drawn to scale, with branch lengths measured in the number of substitutions per site.

The isolate showed multiple resistance to the metals tested, with the organism showing tolerance to all concentrations of Pb (100–1000 mg/L) while the organism tolerated 200 mg/L of Cd and Cr. The tolerance pattern to the tested heavy metals follows the order Pb > Cd = Cr. The growth inhibition curve of strain NWUAB01 on 100 mg/L of each metal at pH 7, agitation of 150 rpm and 25 °C is presented in supplementary Fig. S1. The growth rate of the organism on each of the metal tested is presented in Table S1. The highest optical density (OD) was obtained on the sixth day of growth for each of the metal tested and the control. The optical density increases with time for all metals and control (Fig. S1), with Pb having the least OD of 0.89, followed by Cr with OD of 0.99 after 144 h of growth. Cadmium had the highest OD of 1.24 after the sixth day. In summary, a decrease in the optical density of strain NWUAB01 was observed in the presence of heavy metals compared with the metal-free medium.

### Genomic features of strain NWUAB01

**Figure 3:**
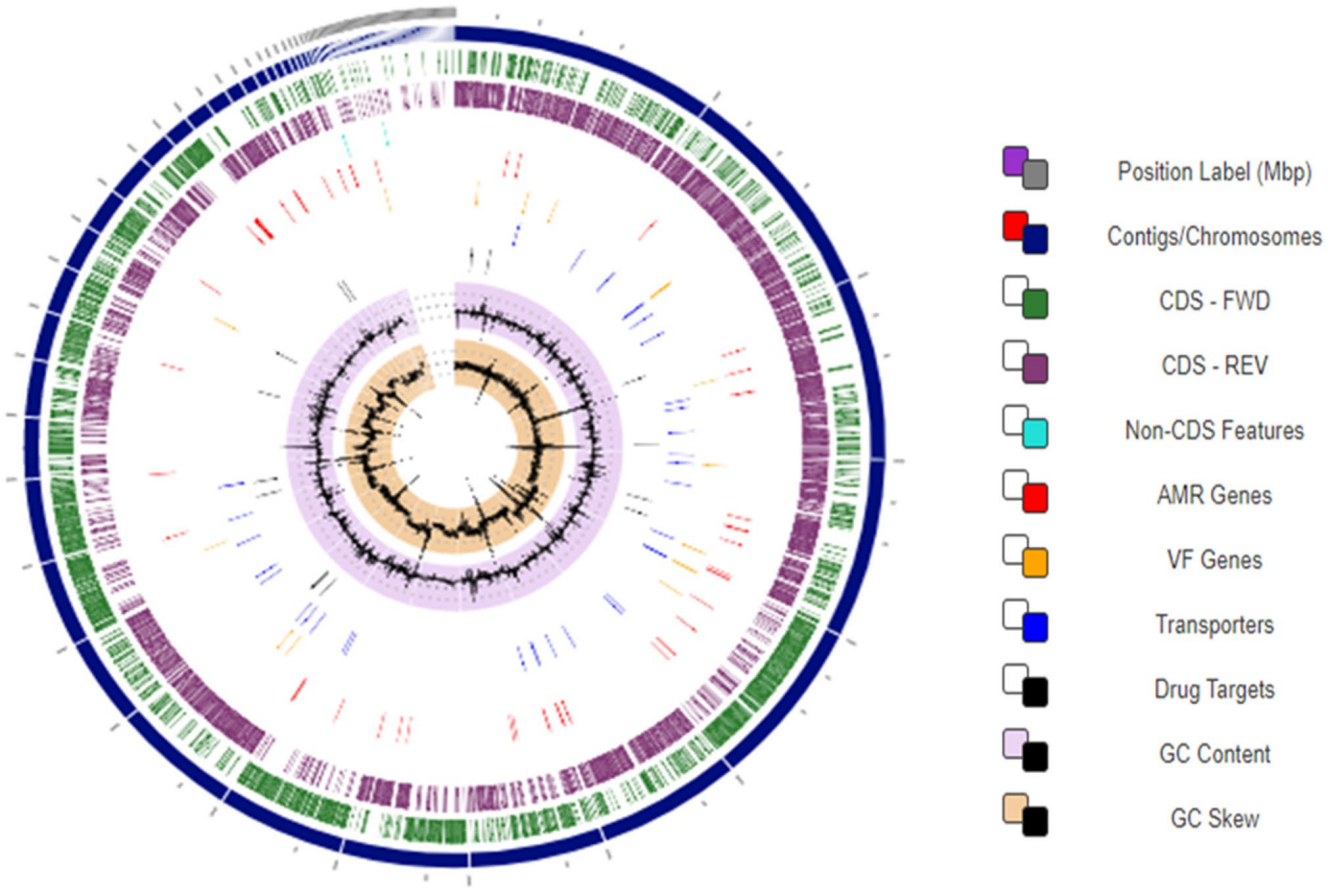
The circular view of the genome of strain NWUAB01 with different features.

The genome of *B. cereus* NWUAB01 was assembled into 91 contigs consisting of 5,989,415 bp and average G + C content of 35.01%. A total of 6306 genes were predicted with 87 tRNA operons and 280 pseudogenes. More features of the genome are presented in Table 1. The circular view of the genome from PATRIC online software is presented in Fig. 3. The circular view showed the contigs, coding and non-coding features, antimicrobial resistance genes, drug targets and the G + C content of the genome (Fig. 3). The RAST annotation categorise the genes into 27 subsystems, with genes for carbohydrate and protein metabolism, amino acid derivatives metabolism, stress response, aromatic compound metabolism, membrane transport, iron acquisition and metabolism, secondary metabolism and several others. The antiSMASH predicted the presence of 15 biosynthetic gene clusters in the genome responsible for secondary metabolite synthesis. The genes predicted include fengycin, lassopeptide, siderophore, bacillibactin, bacteriocin, lanthipeptide amongst others.

**Table 1:** The genomic features of strain NWUAB01.

Gene features	Number/comment
Genes (total)	6306
CDS (total)	6191
Genes (coding)	5911
Genes (RNA)	115
rRNAs	11, 4, 8 (5S, 16S, 23S)
Complete rRNAs	7 (5S)
Partial rRNAs	4, 4, 8 (5S, 16S, 23S)
tRNAs	87
Pseudo genes (total)	280
Pseudo genes (ambiguous residues)	0 of 280
Pseudo genes (frameshift)	128 of 280
Pseudo genes (incomplete)	130 of 280
Pseudo genes (internal stop)	106 of 280
Pseudo genes (multiple problems)	76 of 280

### Heavy metal resistance genes

The amplification of primer-specific heavy metal-resistant genes of chromosomal DNA of strain NWUAB01 yielded amplicons of the expected band size of 600 bp for *cadA,* 398 bp for *CzcD,* and 766 bp for *PbrA* (Fig. 1). No amplification was observed for *CzcA, CzcB, PbrT, chrA*, and *chrB*. This might be as a result of the lack of mechanisms responsible for metal resistance in the genome of the organism. The organism may also use other mechanisms different from the efflux system for metal tolerance.

**Figure 4:**
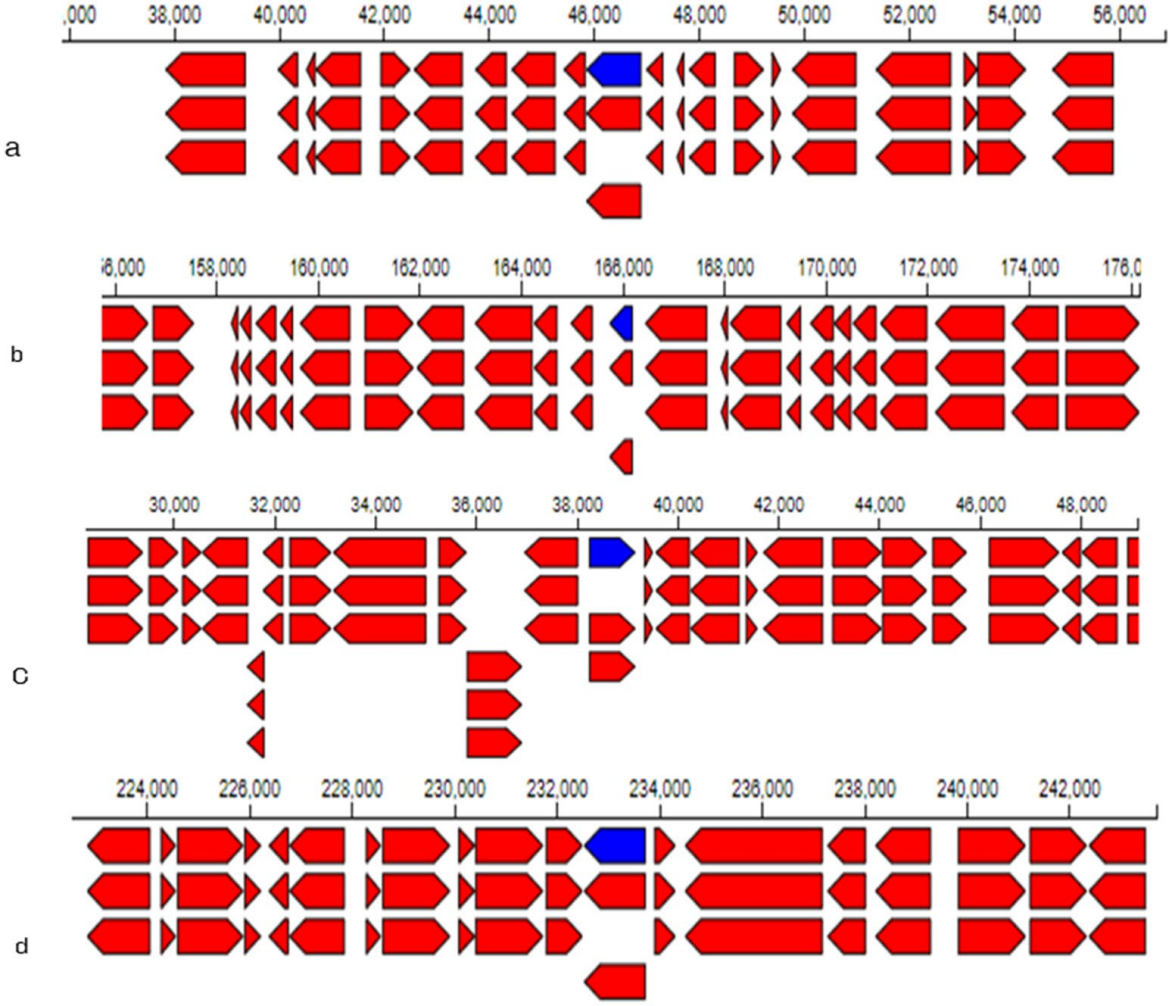
The annotation of heavy metal resistant genes on the genome of strain NWUAB01 and their location on the genome (**a**) arsenic resistance protein (**b**) cadmium resistance transporter (*cad*) (**c**) cobalt–zinc–cadmium resistance protein (*CzcD*) and (**d**) chromate transport protein (*chrA*).

Heavy metal resistance/transport genes are abundant in the genome of strain NWUAB01, which include resistant genes encoding arsenic, cadmium, copper, cobalt and zinc as well as transport genes for chromium, cadmium, lead, magnesium and mercury (Table 2). The annotations of some of these heavy metals and their position on the genome of strain NWUAB01 are presented in Fig. 4 and Fig. S2. The large number of heavy metal-resistant genes in the genome suggests that the organism can tolerate various heavy metals. The genome search against the KEGG database through the RAST server to investigate gene functions and metabolic pathways predicted genes involved in the degradation and metabolism of xenobiotic compounds such as benzoate, fluorobenzoate, fluorine, toluene, biphenyl, naphthalene, anthracene, dichlorobenzene, atrazine, salicylate and styrene.

**Table 2:** Heavy metal resistant and transport genes with their location on the genome of NWUAB01.

Location	Product	Gene	Path way
26787–29154	Cadmium transporting ATPase	*Cad*	Cadmium transport
29177–29555	Cadmium efflux system accessory protein	*Cad*	
165737–166193	Cadmium resistance transporter	*Cad*	
232533–233715	Chromate transport protein	*chrA*	Chromate reduction and transport
54969–55647	Cytoplasmic copper homeostasis protein	*cutC*	Copper resistance transport
7803–9438	Copper resistance protein	*CopC* or *CopD*	
56097–56919	*CorA*, *CorA*-like magnesium transport protein	*CorA*	Magnesium transport
85440–86403	Magnesium transport protein	*CorA*	
38243–39143	Cobalt–zinc–cadmium resistance protein	*CzcD*	Cadmium, cobalt and zinc transport
192511–193837	Arsenic efflux pump protein	*–*	Arsenate reduction and transport
43673–44069	Arsenate reductase family protein	*–*	
45426–45831	Arsenical resistance protein ACR3	*–*	
12198–13494	Manganese transport protein	*MntH*	Manganese transport
12769–14695	Lead, cadmium, zinc and mercury transporting ATPase	*–*	Lead, cadmium, zinc and mercury transport
19421–20372	Zinc ABC transporter, periplasmic binding protein	*ZnuA*	Zinc transport

### Biosurfactant production and characterisation

The genome of strain NWUAB01 revealed the Wzx (O-antigen flippase) and Wzy (oligosaccharide repeat unit polymerase) genes on location 204798–206235 and 203580–204795 respectively (Fig. S3). The genes are responsible for the production of extracellular polymer using the Wzx/Wzy-dependent pathway in strain NWUAB01.

The emulsification of different hydrocarbons and vegetable oil by strain NWUAB01 is presented in Fig. S4 and the biosurfactant properties of the organism are presented in Table 3. The organism was able to haemolyse red blood cells (α-hemolysis), was positive for drop collapse and reduced the surface tension of the growth medium to 39.48 mN/m (Table 3). Strain NWUAB01 produced stable emulsions with various hydrocarbons and vegetable oil with the highest E_24_ of 54% with engine oil and lowest E_24_ of 22% with hexadecane at room temperature and pH 7 (Table 3). The stability of the emulsion produced by strain NWUAB01 was tested at different temperatures and pH (Fig. S5). At 40 °C, the organism gave a stable emulsion with E_24_ of 54%, 30%, 22% and 20% for engine oil, kerosene, hexadecane and vegetable oil respectively (Fig. S5). A reduction in the stability of the emulsion was observed at the extreme temperature and pH. The scanning electron micrograph (SEM) of the surfactant produced by strain NWUAB01 is presented in Fig. S6, which shows the morphology of the surfactant.

**Table 3:** Evaluation of *B. cereus* NWUAB01 for biosurfactant production.

Test		Result
Haemolysis test		Positive
Oil displacement		Negative
Drop collapse		Positive
Surface tension		39.5 ± 0.25 mN/m
Emulsification index (E_24_) (%)	Engine oil	54.0 ± 0.58
	Hexadecane	22.4 ± 0.60
	Kerosene	37.5 ± 0.29
	Vegetable oil	24.0 ± 0.58
Biosurfactant yield		0.38 g/L

Values are means of triplicate readings ± standard error.

### Spectroscopic characterisation

**Figure 5:**
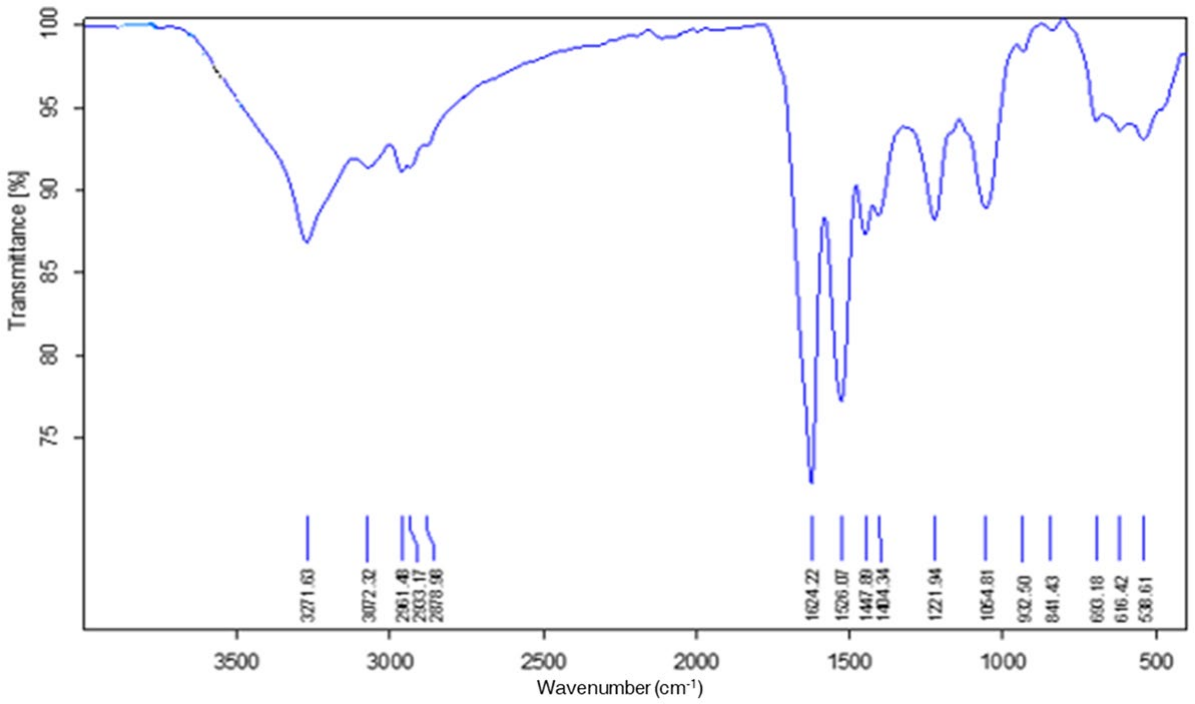
The FTIR spectra of biosurfactant produced by strain NWUAB01.

Fourier transform infrared spectroscopy (FTIR) characterisation of biosurfactant produced by strain NWUAB01 is presented in Fig. 5. The spectrum had a characteristic absorbance band of peptides at 3273 cm^−1^ (stretching mode N–H), 1624 cm^−1^ (stretching mode CO–N) and 1526 cm^−1^ (N–H deformation and C–N stretching mode). The bands obtained at 2961–2878 cm^−1^ and 1624–1447 cm^−1^ represent the presence of aliphatic chains. The absorption around the region 1624 cm^−1^ can be attributed to the lactone carbonyl absorption. The surfactant contains peptide-like moieties. The spectroscopic analysis showed that the biosurfactant is a lipopeptide homolog with different fatty acid chain lenght. The MALDI-TOF spectrum of the surfactant produced by strain NWUAB01 is shown in Fig. S7. There are only well-resolved groups of peaks at m/z values between 182.76 and 696.37.

### Heavy metal removal from contaminated soil

We observed that the biosurfactant was capable of removing 69% of Pb, 54% of Cd and 43% of Cr from the batch experiment from the initial concentration of 100 mg/L (Table S2). The strain removed 83% of Pb, 60% of Cd and 30% of Cr from polluted soil (Table S2). We found that the biosurfactant produced by strain NWUAB01 is efficient in removing metal from contaminated soil. The results of this study showed that Pb has the highest removal followed by Cd and Cr.

## Discussion

Soils polluted with heavy metals are usually sources of organisms resistant to metals^23,24^. Mining soils are rich sources of potential bacterial population resistant to heavy metals, but with reduced bacterial diversity, population size and metabolic activities^25^. Metal resistance might have evolved due to the presence of heavy metals in their growth medium^26^. In this study, soil samples from a gold-mining environment, with natural occurrence of heavy metals, were used for isolating strain NWUAB01, which is consistent with the studies of Oladipo et al.^27^ and Reith et al.^28^ that isolated *B. cereus* from gold mining soil. Thus, the metal-containing environment might have led to the evolvement of mechanisms of resistance to heavy metals in the organism.

Elevated level of tolerance to metals is an important criterion for metal removal by bacterial strains^23^ and strain NWUAB01 showed multiple tolerance to the metals tested and good preliminary metal removal properties, with the ability to grow on all concentrations of Pb tested and on 200 mg/L of Cd and Cr. The organisms showed the ability to withstand varying metal concentrations as reported in different studies^29,30^ from different polluted sites and higher tolerance compared to those observed by Oladipo et al.^27^. Varied responses of strain NWUAB01 to different metal ions observed in this study could be attributed to different modes of action, unique chemistry and level of toxicity of each metal^27,31^.

Multi-metal resistance by microbial strains gives mutual benefits to the single component and is suitable for metal removal^32^. Multi-metal resistance shows various combinations of genetic determinants for metal resistance. This could have probably evolved in the natural environment of the organism. The genetic determinants encode specific metal transport proteins involved in the sequestration of metal ions and regulating active efflux^33^. The resistance pattern of strain NWUAB01 to the tested metals showed that the organism tolerated Pb than Cd and Cr. Many reports have also reported many bacteria with multi-metal resistance abilities^23,34,35^. Multi-metal tolerance in *Bacillus* species has been well documented.

Various mechanisms are employed by microbial cells for metal removal^10^. These include bioaccumulation, biomineralisation, biosorption and biotransformation. The growth kinetics of the organism on different metal revealed that the OD increases with time for all metals and control. The growth rate of strain NWUAB01 on exposure to metal-enriched medium varied with each tested metal. The growth rate was enhanced in the presence of Pb, while there was a reduced growth rate in the presence of Cr. The same pattern of growth rate was observed for Cd and the control. Similarly, the generation time in Pb-medium was lower than that of Cd, Cr and the control. This shows that the doubling time was faster in Pb-medium, which also has a higher number of generations, compared to Cr, Cd and the control. This is an indication that Cd and Cr toxicity to strain NWUAB01 may be dose-dependent^27^. Increased generation time is usually observed for environmental constraint. However, generation time depends on all factors influencing growth, thus growth rate can vary considerably between the different experimental setups. A decrease in the OD of strain NWUAB01 was observed in the presence of heavy metals compared with the metal-free medium. This is similar to the pattern observed by Shim et al.^30^ and Raja et al.^36^. The decrease in the growth of *B. cereus* in the presence of heavy metals might be due to the metal ion interaction with the cell membrane, which increases metal-binding sites and makes it less effective for the transport of materials essential for growth^37^. To understand the mechanism of resistance to metals, growth kinetics is used as an index of adaptation to external constraints^27^. The inverse growth rate relationship observed between metal concentrations and growth rate in tolerant bacteria are characteristics of bacterial growth in response to external stress^38^. The low inhibitory values obtained for Cd and Cr along with a decrease in growth rate in the presence of these metals could be attributed to the decline in efficiency of substrate utilisation as a result of high energy cost of the organism subjected to metal stress^39^.

The presence of extracellular substances, which serve as a barrier in Gram-positive bacteria enhances metal resistance compared to Gram-negative organisms^40^. A direct comparison of metal resistance by strain NWUAB01 with other studies is difficult due to the composition and strength of the medium, the nature of the medium that influences metal bioavailability, complexation, organic constituents, diffusion rate and incubation period, which cause variations in inhibitory concentrations^30,41^.

Genes encoding metal resistance can eliminate or reduce metal toxicity^42^. Hence, strain NWUAB01 was screened for metal-resistant genes. The amplification of primer-specific heavy metal-resistant genes of chromosomal DNA of strain NWUAB01 yielded amplicons of the expected band size for *cadA, CzcD,* and *PbrA*. *cadA*, which is a P-type ATPase, was also found to be present on the organism. *cadA* is cadmium-specific ATPase used for Cd efflux and confers metal resistance to strain NWUAB01. *CzcD* is responsible for the efflux of cobalt, zinc and cadmium. Both *CzcD* and *cadA* operons are energy-dependent efflux systems that confer cadmium resistance^43^. The efflux systems are actively involved in the pumping out of toxic metal ions that enter the cell through ATPase diffusion. *PbrA* is the protein responsible for lead uptake and down-regulation of the metal concentration, which occurs in response to high levels of lead^44^. It thus revealed that isolate NWUAB01 has a functional gene that is key in lead resistance. *PbrA* is an active efflux pump protein that transports Pb ions against the concentration gradient using energy provided by ATP hydrolysis^42^. Metal transport proteins are involved in transporting metal ions outside the cell membrane^45^. These metal transporting proteins are a group of PIB-type ATPases, which governs metal resistance. *cadA, CzcD* and *PbrA* belong to these groups of proteins present in strain NWUAB01 which are involved in metal resistance. These proteins prevent metal accumulation of highly reactive and toxic metals within the cell membrane and play a key role in metal resistance by strain NWUAB01^45^. No amplification was observed for *CzcA, CzcB, PbrT, chrA*, and *chrB*. This might be as a result of the lack of mechanisms responsible for metal resistance in the genetic system of the organism. The organism may also use other mechanisms different from the efflux system for metal tolerance.

However, heavy metal resistance/transport genes are abundant in the genome of *B. cereus* NWUAB01, which include several resistance genes encoding arsenic, cadmium, copper, cobalt and zinc as well as transport genes for chromium, cadmium, lead, magnesium and mercury. The abundant metal-resistant genes in the genome of strain NWUAB01 suggest that the organism can tolerate different metals, which is consistent with a previous report demonstrating the uptake and heavy metal resistance in *B. cereus*^28^. The organism uses different genome-mediated resistance mechanisms such as the transport proteins and efflux pump to survive heavy metal stress. The genome also revealed genes involved in the degradation and metabolism of xenobiotic compounds.

The production of different biosynthetic gene clusters and metabolism of different compounds are adaptive mechanisms for surviving diverse ecological niches^9^, which can be harnessed for different environmental and industrial purposes. The synthesis of biopolymers in bacteria occurs through four pathways namely: ATP-binding cassette transporter-dependent, extracellular synthesis using sucrase protein, Wzx/Wzy-dependent, and synthase-dependent pathways^46,47^. Bacteria using the Wzx/Wzy dependent pathway carry the flippase (Wzx) and polymerase (Wzy) gene in their extracellular polysaccharide operons^47^. The presence of the Wzx (O-antigen flippase) and Wzy (oligosaccharide repeat unit polymerase) gene in the genome of strain NWUAB01 signified the production of extracellular polymer using the Wzx/Wzy-dependent pathway. This pathway produced polymers of various sugar components that results in heteropolysaccharide production^47^.

Blood agar has been used to quantify and screen for biosurfactant production by bacteria^48,49^. Carrillo et al.^50^ and Kumar et al.^49^ found an association between the surfactant production and haemolytic activity, and recommend blood lysis as screening method for the biosurfactant production. Although the lysis of erythrocytes could exclude some biosurfactant producing organisms, it has helped in initial screening of biosurfactant producing organisms. Strain NWUAB01 showed complete haemolysis on erythrocytes and was used as the initial screening test for its selection. The reduction in surface tension of water has been reported in several studies^49,51,52^ for various biosurfactant producing *Bacillus* species. The reduction in surface tension confirmed the production of biosurfactant by strain NWUAB01. The ability to reduce the surface tension of water from 72 to 35 mN/m has been considered as a characteristic of a good surfactant^18^. Strain NWUAB01 has a surface tension that is similar to that produced by *B. cereus* NK1, which has a value of 38 mN/m^51^. It has a better surface tension than *B. cereus*, *B. sphaericus* and *B. fusiformis*, with surface tension of 50, 55.2 and 56.4 mN/m respectively^53^, and *B. amyloliquefaciens* and *B. thuringiensis* with surface tension of 57.7 m/Nm each^54^. Strain NWUAB01 has lower surface tension potential compared to *Bacillus* sp reported by Heryani and Putra^55^, that had a value of 27.1 mN/m. The differences in the surface tension values can be attributed to different production medium, conditions of growth and uniqueness of individual organisms.

The emulsification index is another criterion used in the selection of surface-active-producing bacterial isolates. Satpute et al.^56^ suggested that more than one screening method should be used in the primary screening of potential surface-active agents. Strain NWUAB01 produced stable emulsions with various hydrocarbons and vegetable oil. This appreciable emulsifying property made the organism a suitable surface-active agent. The ability of biosurfactant to emulsify different hydrocarbons and vegetable oil had been reported for *Bacillus* species with different results. Sriram et al.^51^ reported *B. cereus* NK1 to emulsify motor oil, diesel oil, crude oil, petrol and vegetable oil with E_24_ of 80.36%, 55.5%, 70%, 44% and 50.47% respectively. Strain NWUAB01 had lower emulsification index compared to what was reported for *B. cereus* NK1. Likewise, Barakat et al.^54^ reported emulsification index of 60% and 69% with paraffin oil for *B. amyloliquefaciens* and *B. thuringiensis* respectively. This might be as a result of the different components of the production medium and different carbon sources used for producing biosurfactant^57^.

The production of biopolymers that confer resistance to microorganism growing in polluted environments is an important defence mechanism against environmental stress and for survival^20^. Biosurfactants are applied in several fields and their application depends on their stability at different temperatures and pH^58^. Reduction in the stability of the emulsion was observed at the extreme temperature and pH. As the pH increases, there was an increase in the stability of the emulsion until pH 7, after which the stability begins to reduce. The result indicated that an increase in pH had a positive effect on the stability of the emulsion. This could be as a result of the precipitation of biosurfactant at high pH values^58^. Lower stability at reduced pH (< 4) can be attributed to distortion of the biosurfactant structure and precipitation^59^.

The FTIR characterisation of the biosurfactant produced by strain NWUAB01 suggested that the surfactant produced by strain NWUAB01 contained peptide-like moieties, which is typical of lipopeptide surfactants produced by *Bacillus* species described in literature^51,57,60^. The MALDI-TOF spectra of the detected groups could be attributed to the iturin variants as described by Jasim et al.^61^ and Cho et al.^62^. The lack of specific iturin homologs can be attributed to the loss of some of the amino acids such as asparagine, glycine and tyrosine in the structure of iturin, which makes many homologs of the lipopeptide difficult to identify^63^. The composition of the medium of production of lipopeptides can be attributed to some of the variations in the structure^64^. This showed that different compounds could be expressed by *Bacillus* species during changes in growth condition^63^.

Biosurfactant soil washing has been used for metal removal from polluted soils and sediments due to their biodegradability, low toxicity and eco-friendly nature^18,65^. In this study, we evaluated the metal removal capability of biosurfactant produced by strain NWUAB01. We found that the biosurfactant produced by strain NWUAB01 is efficient in removing metal from contaminated soil. In a multi-metal system, the percentage removal of each metal decreases compared to a single metal system. The ability of the biosurfactant to remove metals from contaminated soil was also examined in comparison with strain NWUAB01. We observed that the percentage metal removal was higher for the organism than the surfactant. The results of this study showed that Pb has the highest percentage removal followed by Cd and Cr. This could be due to the affinity of the biosurfactant to different metals^66,67^. The efficiency of metal removal by biosurfactants also depends on the type of biosurfactant and its concentration, soil characteristics and other additives such as acids and bases that may be added^67^. Metal removal efficiency of strain NWUAB01 biosurfactant is higher than those reported by Singh and Cameotra^65^. However, the metal removal efficiency of lipopeptide of marine origin reported by Das et al.^1^ was higher than that of strain NWUAB01. The metal removal potential of strain NWUAB01 corroborated the work of Mulligan et al.^68^, who reported the use of lipopeptide from *B. subtilis* for the removal of Cd, Cu and Zn. Lipopeptides, which are anionic in nature, have better metal sequestration properties and are more effective in metal removal^18^. Removal of metals by biosurfactant has been proposed to occur by surfactant sorption to the soil surface, followed by complexation with metals; thus leading to metal detachment from soil surface by the reduction in the interfacial tension^18^.

In conclusion, the findings in this study showed that strain NWUAB01 is metabolically versatile with high heavy metal affinity that can be harnessed for industrial applications. The presence of diverse metal transport/resistant genes and xenobiotic compounds degradation revealed the ability to survive in varied ecological niches. This study also demonstrated the potential of the biosurfactant produced by strain NWUAB01 for effective removal and recovery of heavy metals for environmental applications.

## Materials and methods

### Isolation and screening of metal-resistant bacterial isolates

Soil samples used in this study were obtained from a gold mining area in Vryburg, South Africa (Table S3 and Fig. S8). The soil samples were collected at a depth of 10–30 cm in triplicate and transported to the laboratory for analysis in sterile plastic bags. Control soil samples were collected few kilometres away from the mine (Table S3 and Fig. S8). The concentration of heavy metals from each sampling site is also presented in the supplementary Table S3. The method of Rajkumar and Freitas^69^ was used in isolating resistant bacteria. Serially diluted soil (1 g) samples were plated on Luria–Bertani (LB) agar supplemented with 50 mg/L of heavy metal solutions (CdSO_4_ (Sigma-Aldrich, India), K_2_CrO_4_ and Pb(NO_3_)_2_, (Sigma-Aldrich, USA)), with each metal at a time^69^. The metal solutions were filter-sterilized through a 0.22 µm filter membrane before they were added to sterile molten LB agar. After that, the plates were incubated for 48 h at 37 °C. Metal-resistant isolates were screened for tolerance to different concentrations (100 to 1000 mg/L) of each heavy metal on LB agar^69^. The organisms were grown on LB agar containing different concentrations of metals from 100 to 1000 mg/L. The plates were incubated for 48 h at 37 °C and observed for growth. Metal sorption by strain NWUAB01 was performed with 100 mg/L of each metal and growth was monitored by measuring OD at 600 nm against the blank at 24 h intervals using a UV spectrophotometer (Thermo Spectronic, Merck, South Africa) as described by Oladipo et al.^27^ with little modification. A 24-h old culture (approximately 10^6^ CFU/mL) in LB broth was used as the inoculum. This was performed in 200 mL Erlenmeyer flask. Briefly, each flask contained a final volume of 100 mL, comprising 98 mL of sterile LB broth, 1 mL of the inoculum and 1 mL of filter sterilized metal solution. Experimental control and blank were also setup. The control comprised 98 mL of LB broth and 1 mL inoculum of the isolate, while the blank contained 98 mL LB broth.

### Identification of strain NWUAB01

Strain NWUAB01 was identified using the following biochemical tests: Gram reaction, sugar fermentation test (fructose, glucose, galactose, lactose, starch, sorbitol, sucrose, maltose, and mannitol), oxidase, catalase test, hydrogen sulphide production, citrate utilisation, methyl red, nitrate reduction, indole production, and Voges–Proskauer test.

The DNA of the isolate was extracted using ZR soil microbe DNA extraction kit (Zymo Research, CA, USA) as described in the manufacturer’s protocol. The quantity and quality of the DNA was determined using NanoDrop Lite spectrophotometer (Thermo Fischer Scientific, CA, USA).

### 16S rRNA characterisation and heavy metal-resistant gene determination

The primer sets used for 16S rRNA gene amplification of strain NWUAB01 are described in the supplementary Table S4. All primer sets were synthesised by Whitehead Scientific, Cape Town, South Africa. A total volume of 25 μL of a reaction mixture of forward and reverse primer (0.5 μL of each), DNA template (1 μL), 2X master mix (12.5 μL) (Biolab, England), and 11 μL nuclease free water was used for the Polymerase Chain Reaction (PCR). PCR was performed using a thermal cycler (Bio-Rad, CA, USA) and the PCR products were analyzed on 1% (w/v) agarose gel supplemented with 10 μL ethidium bromide and electrophoresed. One kilobase molecular marker was used to determine the band size of the amplicons. The amplicons were sequenced at Inqaba Biotech, Pretoria, South Africa.

The sequences obtained were processed and nucleotide BLAST was performed using NCBI GenBank database to determine the evolutionary relatedness of the strain. Molecular Evolutionary Genetics Analysis (MEGAX) software^70^ was used for sequence alignment and the construction of phylogenetic tree. The phylogenetic tree was constructed based on the 16S rDNA using the maximum parsimony method. The sequence was deposited in the NCBI GenBank database.

Strain NWUAB01 was screened for heavy metal resistance genes using primers encoding for chromium (*chrA* and *chrB*), cadmium (*CzcD, CzcB, CzcA*, and *cadA*), and lead (*PbrA* and *PbrT*). The primer sets and their corresponding PCR conditions are presented in Table S4.

### Whole-genome sequencing of strain NWUAB01

Whole-genome sequencing of strain NWUAB01 was performed as described by Babalola et al.^71^. The genome was sequenced on the Illumina Miseq platform. The DNA sample (50 ng) was fragmented by ultrasonication procedure (Covaris), and the fragments selected by size with AMPure XP beads and the ends were repaired. Adapter sequences were ligated to each fragment. The fluorometric method was used for quantification of the fragment and then diluted to a concentration of 4 nM. A MiSeq v3 kit was used for sequencing of the fragments. Genome sequencing of strain NWUAB01 was performed at Inqaba Biotec, Pretoria, South Africa.

The obtained sequences were processed and the quality of the reads were checked using FastQC v.1.0.1 of the KBase platform^72^. The reads were trimmed to filter the low quality and adapter sequences using Trimmomatic v0.36^73^. SPAdes v.3.12.0^74^ was used for de novo assembly. The NCBI Prokaryotic Genome Annotation Pipeline (v4.7)^75^ and Rapid Annotations using Subsystems Technology (RAST v2.0)^76^ were used for genome annotation. Biosynthetic gene clusters were detected with antiSMASH v5.1.0^77^. The circular view of the genome was created using PATRIC v3.5.43^78^.

### Biosurfactant production and characterisation

The pure strain of NWUAB01 was used to quantify the biosurfactant production by different methods, which include haemolytic activity, drop collapse test, oil displacement test, and emulsification activity. All tests were conducted in triplicate. The haemolytic test on blood agar was performed using the method described by Bicca et al.^48^.

The cultivation medium for biosurfactant production contains (g/L): yeast extract, 0.5; sucrose, 5.0; Na_2_HPO_4_·12H_2_O, 1.4; MgSO_4_·7H_2_O, 0.02; KH_2_PO_4_, 0.4 and peptone, 20.0^51^. The medium was seeded with 3% inoculum prepared from LB broth into 50 mL cultivation medium in a 250 mL Erlenmeyer flask and incubated at 37 °C for 7 days at 160 rpm. All readings were taken with supernatant obtained by centrifuging the cultures at 10,000 rpm for 20 min.

The drop collapse test was determined as described by Sriram et al.^51^. Mineral oil (2 μL) was added to each well of a 96-well microtiter plate and allowed to equilibrate for one hour at 37 °C. After that, 5 μL of the culture supernatant was added to the centre of each well over the oil layer. After one minute, the shape of the oil drop was examined. A flattened drop was recorded as positive for biosurfactant production. Water was used as a negative control.

The oil displacement test was performed as described by Sriram et al.^51^. A Petri dish (150 mm diameter) was filled with 40 mL sterile distilled water and engine oil (15 μL) was added. After that, 10 μL of the supernatant was added to the centre of the oil film and the halo zone was measured after 30 s of incubation.

The emulsification activity of strain NWUAB01 was determined by measuring the emulsification index (E_24_) after 24 h. A 2 mL volume of the culture supernatant was added to 2 mL of kerosene in a test tube and the mixture was vortexed at high speed for 2 min. The E_24_ was calculated as the percentage of the height of emulsified layer divided by the total height of the liquid column^20^. The test was also performed using engine oil, hexadecane and vegetable oil in place of kerosene. The stability of the emulsion produced was determined at different temperatures and pH. Surface tension of the supernatant was measured at room temperature with a force tensiometer (Sigma 702, Biolin Scientific, Sweden) using the du Nouy ring method.

The biosurfactant produced was extracted and purified as described by Gond et al.^79^. Strain NWUAB01 was grown in 1 L cultivation medium for 7 days at 37 °C at 200 rpm. The cell free supernatant obtained by centrifuging at 5000 rpm for 15 min at 4 °C was precipitated by adding HCl to reduce the pH to 2 and incubated overnight at 4 °C. The precipitate was collected by centrifugation at 10,000 rpm for 15 min at 4 °C and dissolved in methanol and then filtered using a membrane filter (0.22 µm PTFE) to remove cell components and large particles. The resulting mixture was then concentrated using a vacuum evaporator at 30 °C and lyophilised. The lyophilised biosurfactant was characterised by FTIR. The spectrum was collected from 400 to 4000 wavenumbers (cm^−1^) with a resolution of 4 cm^−1^ at an average of 32 scans using an Alpha II Platinum-ATR IR spectrometer (Brucker, USA). The lyophilised biosurfactant was also subjected to scanning electron microscope (JSM-6390LV, JEOL, Japan). The molecular mass of the surfactant was determined using Micromass ToFSpec matrix-assisted laser desorption ionization time-of-flight mass spectrometry (MICRO-TOF-MS). Applied Biosystems 4800 Plus MICRO-TOF/TOF analyzer (AB Sciex, USA) was used to obtain a purified sample. The analyzer was operated in the positive ion mode with 337 nm nitrogen laser for ionization, accelerating voltage of 20 kV and a-cyano-4-hydroxycinnamic acid for matrix. The molecular weight was determined by mass spectrum smart formula tools and the mass spectrometry was determined using the Bruker compass data analysis.

### Remediation of heavy metal-contaminated soil with biosurfactant

Biosurfactant washing of heavy metal-polluted soil was performed in a batch experiment as described by Singh and Cameotra^65^. A gram of heavy metal (100 mg/L of each metal salt) contaminated soil sample was placed in a 50 mL centrifuge tube with 25 mL of 0.5 g/L of purified biosurfactant. The soil samples used were uncontaminated soil samples supplemented with 100 mg/L of each metal salt. The experiment was performed at room temperature and pH 7. The mixture was centrifuged at 5000 rpm for 15 min and the supernatant was filtered. The metal composition of the filtered supernatant was analyzed by ICP-OE Spectrometry (Agilent Technologies, Palo Alto, CA, USA). The positive control used is strain NWUAB01, while sterile distilled water served as the negative control. The metal removal efficiency was calculated using the formula:

$$Removal rate \left(\%\right)=\frac{Ci -Ce }{Ci} \times 100$$

where C_i_ and C_e_ are the initial and final concentrations of each metal respectively.

### Ethical approval

This article does not contain any studies with human participants or animals performed by any of the authors.

## Supplementary information

Supplementary Information

## Data Availability

*B. cereus* NWUAB01 has been deposited at the NCBI database under the 16S rRNA gene accession number MH399230 and whole genome accession number QNGD00000000 and BioProject number PRJNA476495. The Sequence Read Archive raw reads are deposited under accession number SRR7647568.

## References

[1] Das P, Mukherjee S, Sen R (2009). Biosurfactant of marine origin exhibiting heavy metal remediation properties. Bioresour. Technol..

[2] Nagajyoti P, Lee K, Sreekanth T (2010). Heavy metals, occurrence and toxicity for plants: a review. Environ. Chem. Lett..

[3] Chibuike G, Obiora S (2014). Heavy metal polluted soils: effect on plants and bioremediation methods. Appl. Environ. Soil Sci..

[4] Barakat M (2011). New trends in removing heavy metals from industrial wastewater. Arab. J. Chem..

[5] Ayangbenro AS, Babalola OO (2017). A new strategy for heavy metal polluted environments: a review of microbial biosorbents. Int. J. Environ. Res. Public Health.

[6] Cervantes C (2001). Interactions of chromium with microorganisms and plants. FEMS Microbiol. Rev..

[7] Wuana RA, Okieimen FE (2011). Heavy metals in contaminated soils: a review of sources, chemistry, risks and best available strategies for remediation. ISRN Ecol..

[8] Voica DM, Bartha L, Banciu HL, Oren A (2016). Heavy metal resistance in halophilic bacteria and archaea. FEMS Microbiol. Lett..

[9] Nahurira R (2019). In silico genome analysis reveals the metabolic versatility and biotechnology potential of a halotorelant phthalic acid esters degrading *Gordonia alkanivorans* strain YC-RL2. AMB Express.

[10] Lin CC, Lin HL (2005). Remediation of soil contaminated with the heavy metal (Cd^2+^). J. Hazard. Mater..

[11] Fomina M, Gadd GM (2014). Biosorption: current perspectives on concept, definition and application. Bioresour. Technol..

[12] Wu G (2010). A critical review on the bio-removal of hazardous heavy metals from contaminated soils: issues, progress, eco-environmental concerns and opportunities. J. Hazard. Mater..

[13] Pal A, Paul A (2008). Microbial extracellular polymeric substances: central elements in heavy metal bioremediation. Indian J. Microbiol..

[14] Li K, Pidatala VR, Shaik R, Datta R, Ramakrishna W (2014). Integrated metabolomic and proteomic approaches dissect the effect of metal-resistant bacteria on maize biomass and copper uptake. Environ. Sci. Technol..

[15] Ayangbenro AS, Babalola OO (2018). Metal(loid) bioremediation: strategies employed by microbial polymers. Sustainability.

[16] Valls M, De Lorenzo V (2002). Exploiting the genetic and biochemical capacities of bacteria for the remediation of heavy metal pollution. FEMS Microbiol. Rev..

[17] Singh P, Cameotra SS (2004). Enhancement of metal bioremediation by use of microbial surfactants. Biochem. Biophys. Res. Commun..

[18] Mulligan CN (2005). Environmental applications for biosurfactants. Environ. Pollut..

[19] Dahrazma B, Mulligan CN (2007). Investigation of the removal of heavy metals from sediments using rhamnolipid in a continuous flow configuration. Chemosphere.

[20] Rizzo C (2015). Biosurfactant activity, heavy metal tolerance and characterization of *Joostella* strain A8 from the Mediterranean polychaete *Megalomma claparedei* (Gravier, 1906). Ecotoxicology.

[21] Cáliz J (2013). Emerging resistant microbiota from an acidic soil exposed to toxicity of Cr, Cd and Pb is mainly influenced by the bioavailability of these metals. J. Soils Sed..

[22] Hasegawa M, Kishino H, Yano T-A (1985). Dating of the human-ape splitting by a molecular clock of mitochondrial DNA. J. Mol. Evol..

[23] Ndeddy Aka RJ, Babalola OO (2017). Identification and characterization of Cr-, Cd-, and Ni-tolerant bacteria isolated from mine tailings. Bioremediat. J..

[24] Xie Y (2016). Effect of heavy metals pollution on soil microbial diversity and bermudagrass genetic variation. Front. Plant Sci..

[25] Zampieri BDB, Pinto AB, Schultz L, de Oliveira MA, de Oliveira AJFC (2016). Diversity and distribution of heavy metal-resistant bacteria in polluted sediments of the Araça Bay, São Sebastião (SP), and the relationship between heavy metals and organic matter concentrations. Microb. Ecol..

[26] Çolak F, Atar N, Yazıcıoğlu D, Olgun A (2011). Biosorption of lead from aqueous solutions by *Bacillus* strains possessing heavy-metal resistance. Chem. Eng. J..

[27] Oladipo OG (2018). Tolerance and growth kinetics of bacteria isolated from gold and gemstone mining sites in response to heavy metal concentrations. J. Environ. Manag..

[28] Reith F, McPhail D, Christy A (2005). *Bacillus cereus*, gold and associated elements in soil and other regolith samples from Tomakin Park Gold Mine in southeastern New South Wales, Australia. J. Geochem. Explor..

[29] Gnanamani A (2010). Microbial products (biosurfactant and extracellular chromate reductase) of marine microorganism are the potential agents reduce the oxidative stress induced by toxic heavy metals. Colloids Surf. B. Biointerfaces.

[30] Shim J, Babu AG, Velmurugan P, Shea PJ, Oh B-T (2014). *Pseudomonas fluorescens* JH 70–4 promotes Pb stabilization and early seedling growth of Sudan grass in contaminated mining site soil. Environ. Technol..

[31] Edwards SJ, Kjellerup BV (2013). Applications of biofilms in bioremediation and biotransformation of persistent organic pollutants, pharmaceuticals/personal care products, and heavy metals. Appl. Microbiol. Biotechnol..

[32] Alisi C (2009). Bioremediation of diesel oil in a co-contaminated soil by bioaugmentation with a microbial formula tailored with native strains selected for heavy metals resistance. Sci. Tot. Environ..

[33] Pal A, Dutta S, Mukherjee P, Paul A (2005). Occurrence of heavy metal-resistance in microflora from serpentine soil of Andaman. J. Basic Microbiol..

[34] Abou-Shanab R, Van Berkum P, Angle J (2007). Heavy metal resistance and genotypic analysis of metal resistance genes in Gram-positive and Gram-negative bacteria present in Ni-rich serpentine soil and in the rhizosphere of *Alyssum murale*. Chemosphere.

[35] Dell’Amico E, Mazzocchi M, Cavalca L, Allievi L, Andreoni V (2008). Assessment of bacterial community structure in a long-term copper-polluted ex-vineyard soil. Microbiol. Res..

[36] Raja CE, Anbazhagan K, Selvam GS (2006). Isolation and characterization of a metal-resistant *Pseudomonas aeruginosa* strain. World J. Microbiol. Biotechnol..

[37] Achal V, Pan X, Fu Q, Zhang D (2012). Biomineralization based remediation of As (III) contaminated soil by *Sporosarcina ginsengisoli*. J. Hazard. Mater..

[38] Bloem J, Breure AM, Markert BA, Breure AM, Zechmeister HG (2003). Trace Metals and Other Contaminants in the Environment.

[39] Giller KE, Witter E, McGrath SP (2009). Heavy metals and soil microbes. Soil Biol. Biochem..

[40] Ianeva OD (2009). Mechanisms of bacteria resistance to heavy metals. Mikrobiol. Z..

[41] Govarthanan M (2013). Significance of autochthonous *Bacillus* sp. KK1 on biomineralization of lead in mine tailings. Chemosphere.

[42] Shin M-N (2012). Characterization of lead resistant endophytic *Bacillus* sp. MN3–4 and its potential for promoting lead accumulation in metal hyperaccumulator *Alnus firma*. J. Hazard. Mater..

[43] Nies DH, Nies A, Chu L, Silver S (1989). Expression and nucleotide sequence of a plasmid-determined divalent cation efflux system from *Alcaligenes eutrophus*. Proc. Natl. Acad. Sci..

[44] Wu W (2016). Genome sequencing reveals mechanisms for heavy metal resistance and polycyclic aromatic hydrocarbon degradation in *Delftia lacustris* strain LZ-C. Ecotoxicology.

[45] Naik MM, Dubey SK (2013). Lead resistant bacteria: lead resistance mechanisms, their applications in lead bioremediation and biomonitoring. Ecotoxicol. Environ. Saf..

[46] Kumar M, Kumar M, Pandey A, Thakur IS (2019). Genomic analysis of carbon dioxide sequestering bacterium for exopolysaccharides production. Sci. Rep..

[47] Schmid J, Sieber V, Rehm B (2015). Bacterial exopolysaccharides: biosynthesis pathways and engineering strategies. Front. Microbiol..

[48] Bicca FC, Fleck LC, Ayub MAZ (1999). Production of biosurfactant by hydrocarbon degrading *Rhodococcus ruber* and *Rhodococcus erythropolis*. Rev. Microbiol..

[49] Kumar AP (2016). Evaluation of orange peel for biosurfactant production by *Bacillus licheniformis* and their ability to degrade naphthalene and crude oil. 3 Biotech.

[50] Carrillo P, Mardaraz C, Pitta-Alvarez S, Giulietti A (1996). Isolation and selection of biosurfactant-producing bacteria. World J. Microbiol. Biotechnol..

[51] Sriram MI (2011). Biofilm inhibition and antimicrobial action of lipopeptide biosurfactant produced by heavy metal tolerant strain *Bacillus cereus* NK1. Colloids Surf. B. Biointerfaces.

[52] Tuleva B, Christova N, Jordanov B, Nikolova-Damyanova B, Petrov P (2005). Naphthalene degradation and biosurfactant activity by *Bacillus cereus* 28BN. Z. Naturforsch. C.

[53] Bento FM, de Oliveira Camargo FA, Okeke BC, Frankenberger WT (2005). Diversity of biosurfactant producing microorganisms isolated from soils contaminated with diesel oil. Microbiol. Res..

[54] Barakat KM, Hassan SW, Darwesh OM (2017). Biosurfactant production by haloalkaliphilic *Bacillus* strains isolated from Red Sea, Egypt. Egypt. J. Aquat. Res..

[55] Heryani H, Putra MD (2017). Kinetic study and modeling of biosurfactant production using *Bacillus* sp. Electron. J. Biotechnol..

[56] Satpute S, Bhawsar B, Dhakephalkar P, Chopade B (2008). Assessment of different screening methods for selecting biosurfactant producing marine bacteria. Indian J. Mar. Sci..

[57] Pereira JF (2013). Optimization and characterization of biosurfactant production by *Bacillus subtilis* isolates towards microbial enhanced oil recovery applications. Fuel.

[58] Khopade A (2012). Production and stability studies of the biosurfactant isolated from marine *Nocardiopsis* sp. B4. Desalination.

[59] Zou C (2014). Characterization and optimization of biosurfactants produced by *Acinetobacter baylyi* ZJ2 isolated from crude oil-contaminated soil sample toward microbial enhanced oil recovery applications. Biochem. Eng. J..

[60] Rivardo F, Turner R, Allegrone G, Ceri H, Martinotti M (2009). Anti-adhesion activity of two biosurfactants produced by *Bacillus* spp. prevents biofilm formation of human bacterial pathogens. Appl. Microbiol. Biotechnol..

[61] Jasim B, Sreelakshmi K, Mathew J, Radhakrishnan E (2016). Surfactin, iturin, and fengycin biosynthesis by endophytic *Bacillus* sp. from *Bacopa monnieri*. Microb. Ecol..

[62] Cho S-J, Lee SK, Cha BJ, Kim YH, Shin K-S (2003). Detection and characterization of the *Gloeosporium gloeosporioides* growth inhibitory compound iturin A from *Bacillus subtilis* strain KS03. FEMS Microbiol. Lett..

[63] Chen H, Chen Z, Zhang Y, Zhu W (2014). Identification of antifungal peptides from *Bacillus subtilis* Bs-918. Anal. Lett..

[64] Akpa E (2001). Influence of culture conditions on lipopeptide production by *Bacillus* subtilis. Appl. Biochem. Biotechnol..

[65] Singh AK, Cameotra SS (2013). Efficiency of lipopeptide biosurfactants in removal of petroleum hydrocarbons and heavy metals from contaminated soil. Environ. Sci. Pollut. Res..

[66] Ochoa-Loza FJ, Noordman WH, Jannsen DB, Brusseau ML, Maier RM (2007). Effect of clays, metal oxides, and organic matter on rhamnolipid biosurfactant sorption by soil. Chemosphere.

[67] da Rocha Junior RB (2019). Application of a low-cost biosurfactant in heavy metal remediation processes. Biodegradation.

[68] Mulligan CN, Yong RN, Gibbs BF (2001). Heavy metal removal from sediments by biosurfactants. J. Hazard. Mater..

[69] Rajkumar M, Freitas H (2008). Influence of metal resistant-plant growth-promoting bacteria on the growth of *Ricinus communis* in soil contaminated with heavy metals. Chemosphere.

[70] Kumar S, Stecher G, Li M, Knyaz C, Tamura K (2018). MEGA X: molecular evolutionary genetics analysis across computing platforms. Mol. Biol. Evol..

[71] Babalola OO, Aremu BR, Ayangbenro AS (2019). Draft genome sequence of heavy metal-resistant *Bacillus cereus* NWUAB01. Microbiol. Resour. Announc..

[72] 72Arkin, A. P. *et al.* The DOE systems biology knowledgebase (KBase). *bioRxiv*, 096354 (2016).

[73] Bolger AM, Lohse M, Usadel B (2014). Trimmomatic: a flexible trimmer for Illumina sequence data. Bioinformatics.

[74] Nurk S (2013). Assembling single-cell genomes and mini-metagenomes from chimeric MDA products. J. Comput. Biol..

[75] Haft DH (2017). RefSeq: an update on prokaryotic genome annotation and curation. Nucleic Acids Res..

[76] Aziz RK (2008). The RAST server: rapid annotations using subsystems technology. BMC Genom..

[77] Blin K (2019). antiSMASH 5.0: updates to the secondary metabolite genome mining pipeline. Nucleic Acids Res..

[78] Wattam AR (2016). Improvements to PATRIC, the all-bacterial bioinformatics database and analysis resource center. Nucleic Acids Res..

[79] Gond SK, Bergen MS, Torres MS, White JF (2015). Endophytic *Bacillus* spp. produce antifungal lipopeptides and induce host defence gene expression in maize. Microbiol. Res..

