# Supplementary material for: Genomic analysis of Bacillus cereus NWUAB01 and its heavy metal removal from polluted soil
Source: Sci Rep. 2020 Nov 12;10:19660. doi: 10.1038/s41598-020-75170-x (PMC7665202; doi:10.1038/s41598-020-75170-x)
Supplement: Supplementary file 1 — Supplementary Information [file 41598_2020_75170_MOESM1_ESM.pdf]

Genomic analysis of *Bacillus cereus* NWUAB01 and its heavy metal removal from polluted soil

Ayansina Segun Ayangbenro and Olubukola Oluranti Babalola*

Food Security and Safety, Faculty of Natural and Agricultural Sciences, North-West University, Private Bag X2046, Mmabatho 2735, South Africa.

***Correspondence: Olubukola.Babalola@nwu.ac.za; +27183892568**

Table S1. The growth rate of strain NWUAB01 on each of the metal tested and the control

	Generation time	Number of generations	Specific growth rate
Cd	88.87	0.27	0.01
Cr	93.67	0.26	0.01
Pb	61.34	0.39	0.01
Control	88.87	0.27	0.01

Table S2. Heavy metal removal by strain NWUAB01 and its biosurfactant

Metal	Biosurfactant	Strain NWUAB01
	Percentage removal (%)	Percentage removal (%)
Cd	54.30	60.90
Cr	43.52	30.85
Pb	69.78	83.83
Multi-metal		
Cd	40.13	45.80
Cr	30.45	33.72
Pb	65.23	72.39

Table S3. The sampling sites and their corresponding metal concentration

Sampling site	Location	Cd (mg/kg)	Cr (mg/kg)	Pb (mg/kg)	Zinc (mg/kg)
1	26° 10' 5.27" S<br>25° 14' 47.69" E	0.26±0.00[b]	0.35±0.00 [c]	1.86±0.00 [b]	0.96±0.00 [b]
2	26° 10' 14.28" S<br>25° 14' 14.58" E	0.02±0.00 [d]	0.27±0.01 [f]	0.23±0.01 [de]	0.3±0.02 [c]
3	26° 10' 3.19" S<br>25° 15' 19.47" E	0.04±0.00 [c]	0.37±0.00 [b]	0.3±0.01 [c]	0.16±0.00 [d]
4	26° 10' 2.18" S<br>25° 15' 20.66" E	0.36±0.00 [a]	0.41±0.00 [a]	2.4±0.04 [a]	1.2±0.00 [a]
5	26° 11' 11.03" S<br>25° 15' 20.66" E	0.03±0.00 [d]	0.29±0.00 [e]	0.21±0.00 [de]	0.07±0.00 [g]
6	26° 11' 11.01" S<br>25° 15' 40.87" E	0.02±0.00 [d]	0.22±0.00 [g]	0.2±0.00 [e]	0.07±0.00 [g]
7	26° 9' 40.15" S<br>25° 14' 55.40" E	0.02±0.00 [d]	0.27±0.00 [f]	0.25±0.00 [cd]	0.14±0.00 [e]
8 (Control)	26° 6' 42.59" S<br>25° 9' 24.52" E	0.03±0.00 [c]	0.33±0.00 [d]	0.28±0.00 [c]	0.11±0.00 [f]

Values are means of triplicate readings ± standard error
Values with the same letter within a column are not significantly different ($P < 0.05$)

Table S4. The sequences of the primer sets used for amplification of 16S rRNA and heavy metal-resistant genes

Primer	Primer sequence	Expected band size (bp)	Reference
16S rRNA	F- AGAGTTTGATCCTGGCTCAG<br>R- ACGGCTACCTTGTTACGACTT	1,500	Marin, et al. [1]
CzcA	F- GTTTGAACGTATCATTAGTTTC<br>R - GTAGCCATCCGAAATATTCG	1,885	Nies, et al. [2]
CzcD	F- CAGGTCACTGACACGACCAT<br>R- CATGCTGATGAGATTGATGATC	398	Nies, et al. [2]
CzcB	F- CTATTTCGAACAAACAAAAGG<br>R- CTTCAGAACAAAACTGTTGG	1,520	Abou-Shanab, et al. [3]
PbrT	F- ATGGTGATTGCTTTAGTT<br>R- TTAGGCTTGCTTCTTTTT		Shin, et al. [4]
PbrA	F- ATGAGCGAATGTGGCTCGAAG<br>R- TCATCGACGCAACAGCCTCAA	766	Shin, et al. [4]
*chr*A	F- CTTATACGCTACGCCAACTG<br>R- GTAATGGCATTCAGTCGCTTG	1,292	Nies, et al. [5]
chrB	F- GTCGTTAGCTTGCCAACATC<br>R- CGAAAGCAAGATGTCGATCG	450	Nies, et al. [5]
cadA	F- GACAAGACYGGMACYMTCAC<br>R- GCRTGGTTRATSCGTC	600	Dell'Amico, et al. [6]

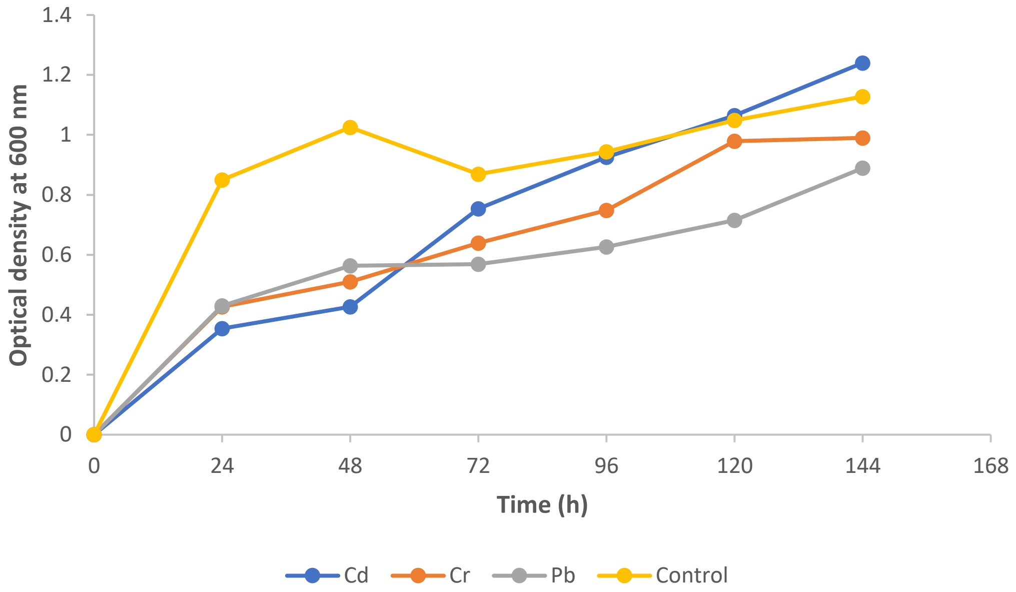

Fig. S1 Time course growth of *B. cereus* NWUAB01 on different heavy metals

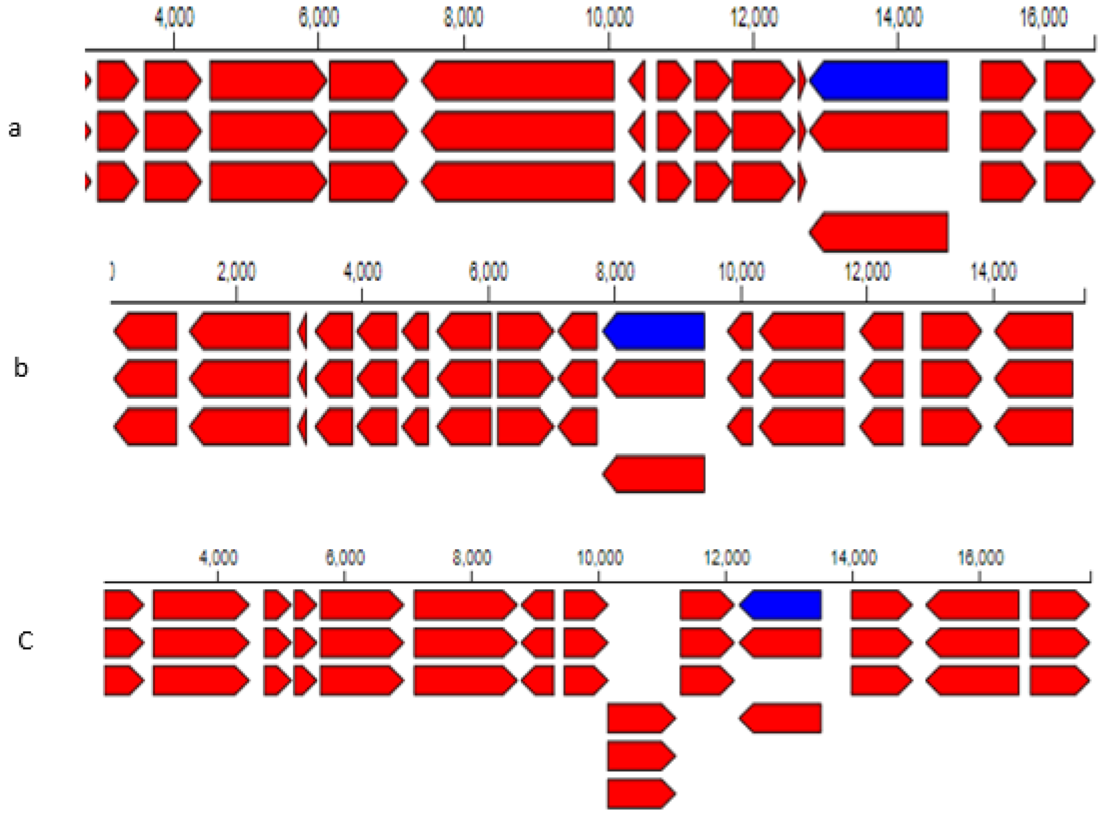

Fig. S2 The annotation for (a) Lead, cadmium, zinc and mercury transporting ATPase (b) copper resistance protein and (c) manganese transport protein

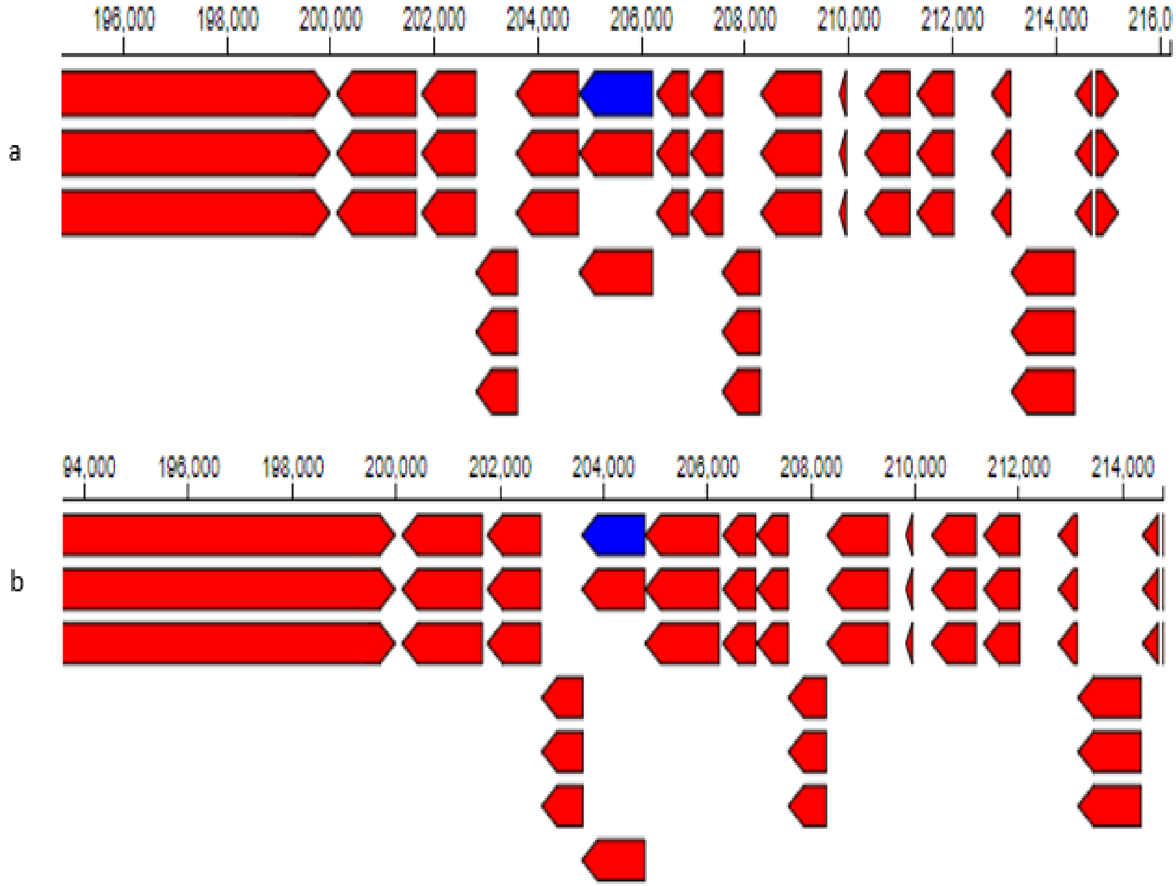

Fig. S3 Gene annotation of the (a) Wzx and (b) Wzy and their respective locus on the genome of strain NWUAB01

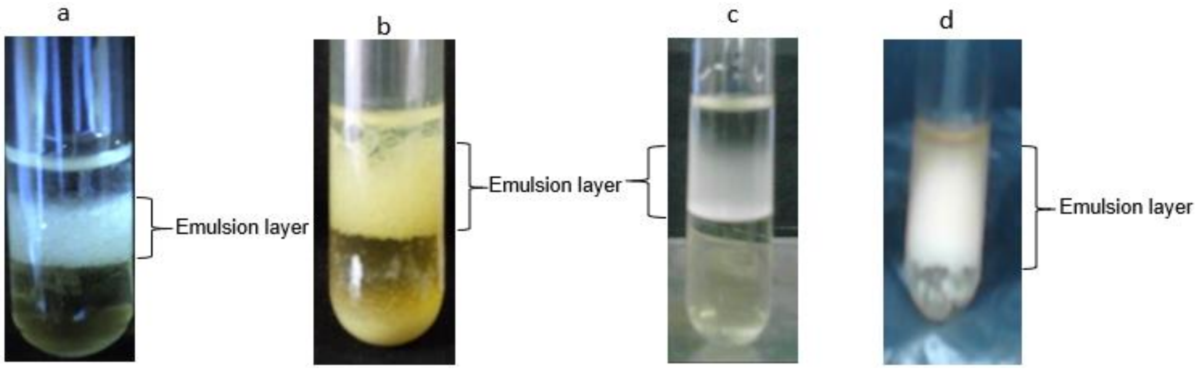

Fig. S4 Emulsification of (a) kerosene (b) kerosene in the presence of heavy metals (c) vegetable oil (d) engine oil by strain NWUAB01

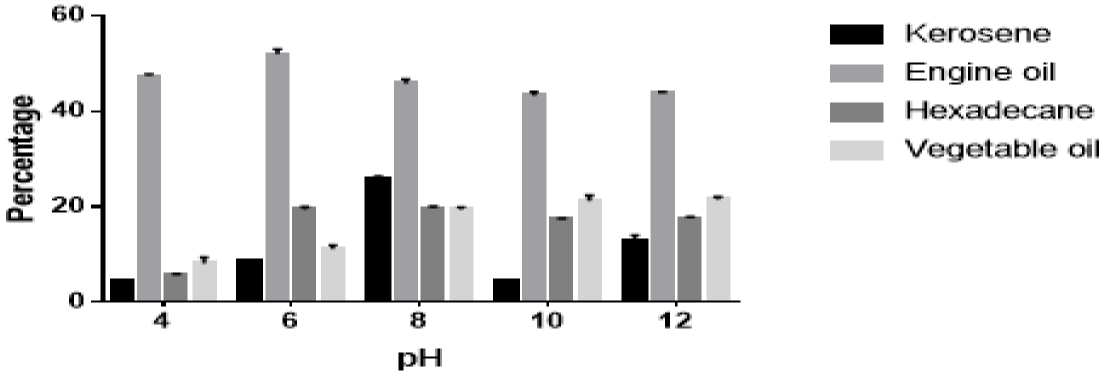

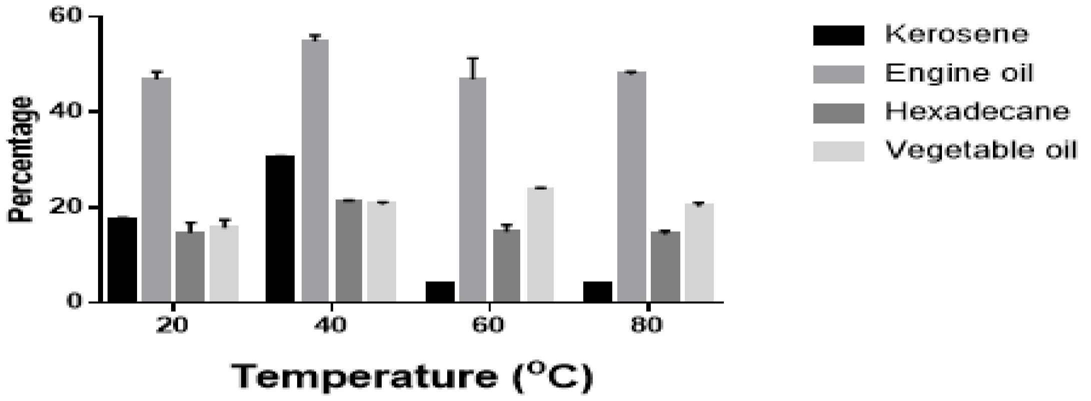

Fig. S5 Stability of the emulsion produced by strain NWUAB01 at different pH and temperature

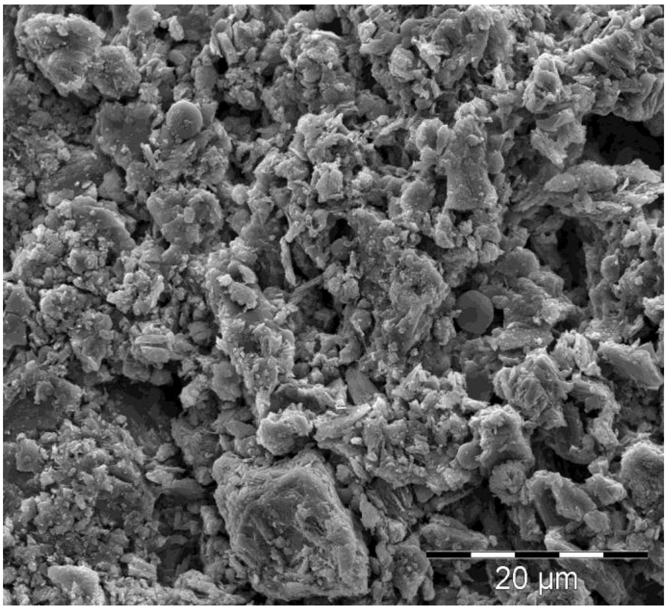

Fig. S6 The SEM image of the biosurfactant produced be strain NWUAB01

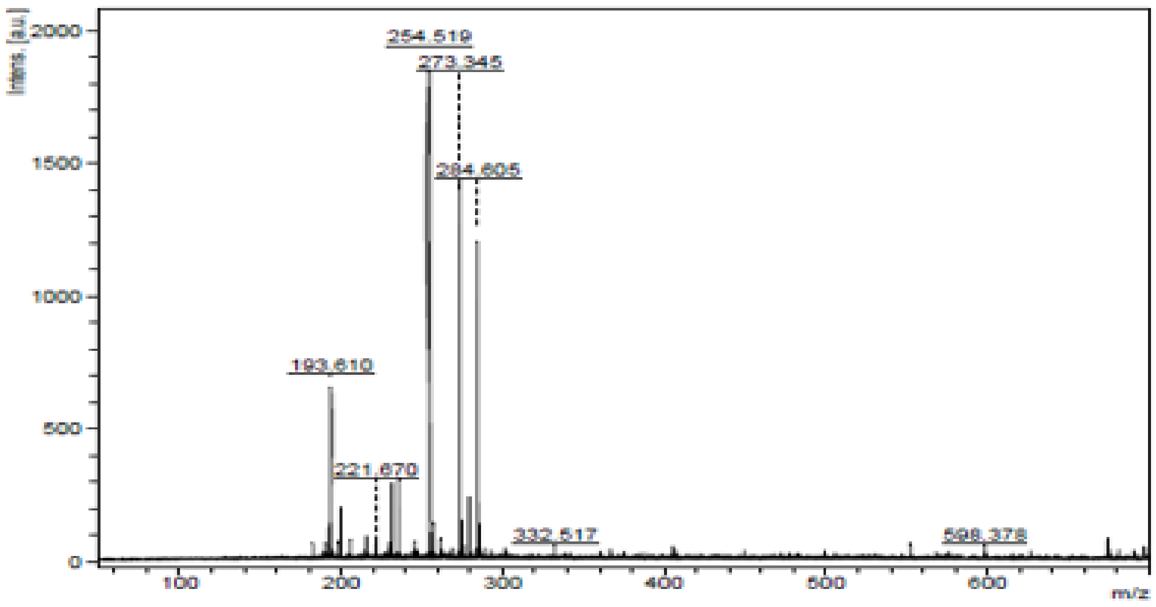

Fig. S7 The mass spectrum of the purified biosurfactant produced by strain NWUAB01

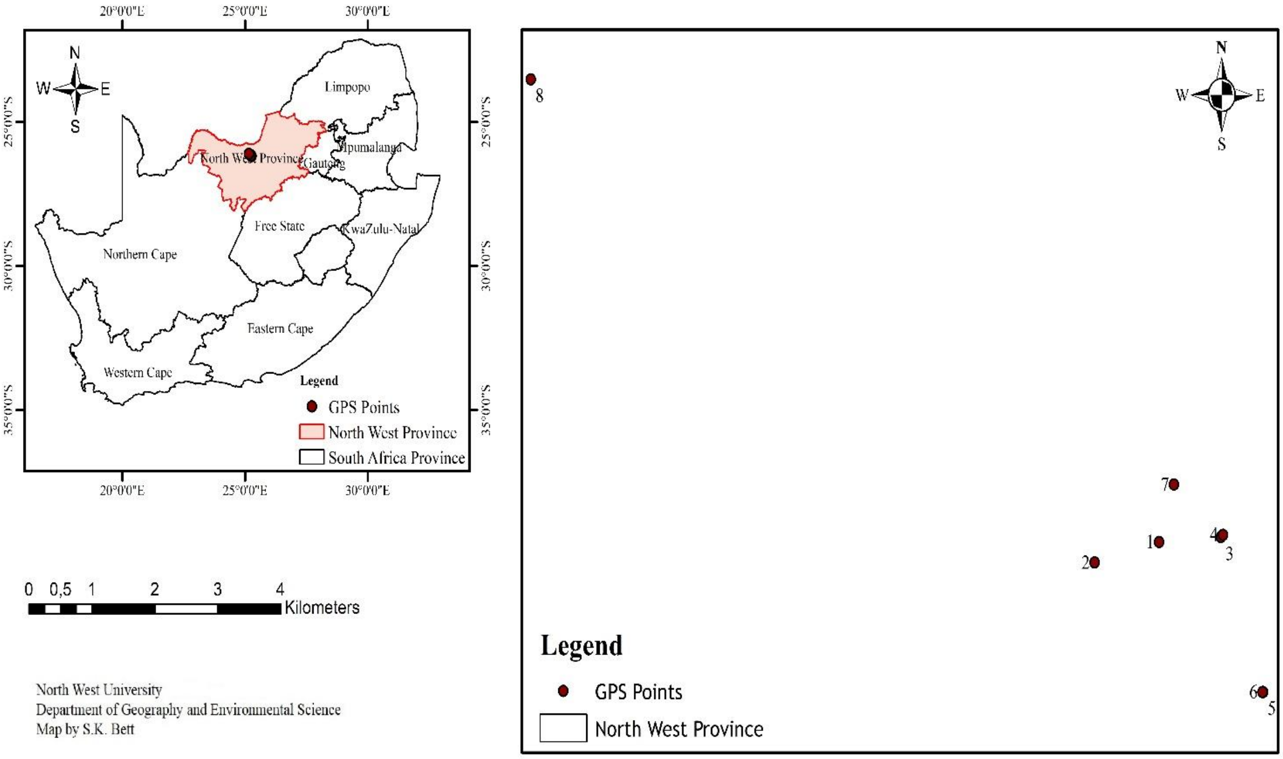

Fig. S8 The map showing the sampling sites. The map was generated using ArcMap (version 10.5.1) (https://desktop.arcgis.com/en/arcmap/)

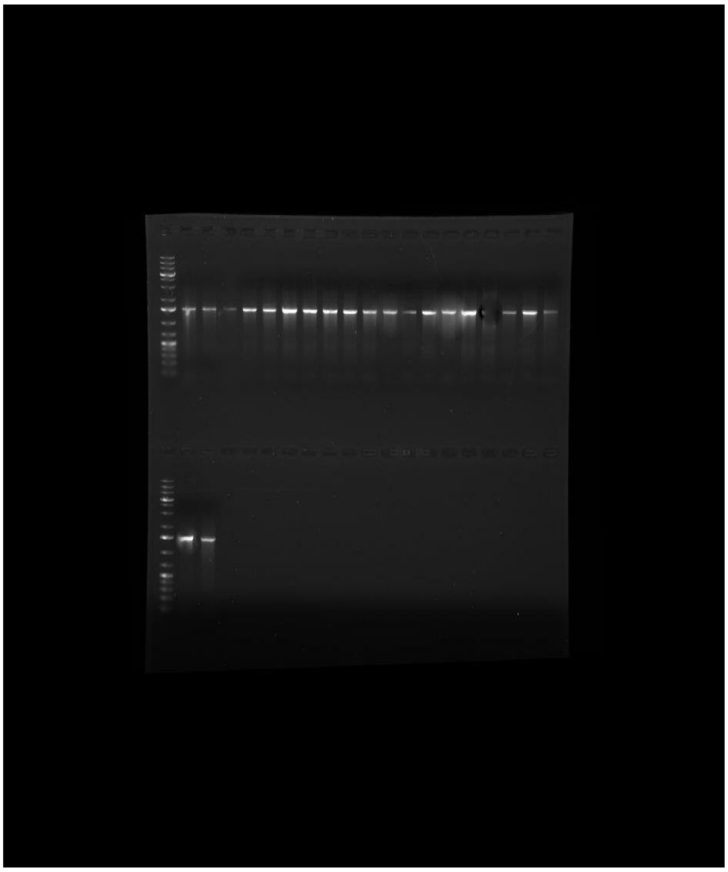

Gel electrophoresis of the 16S rRNA gene of bacterial isolates from mining soil. It shows the predicted 1,500 bp amplicons of the isolates and negative for the control

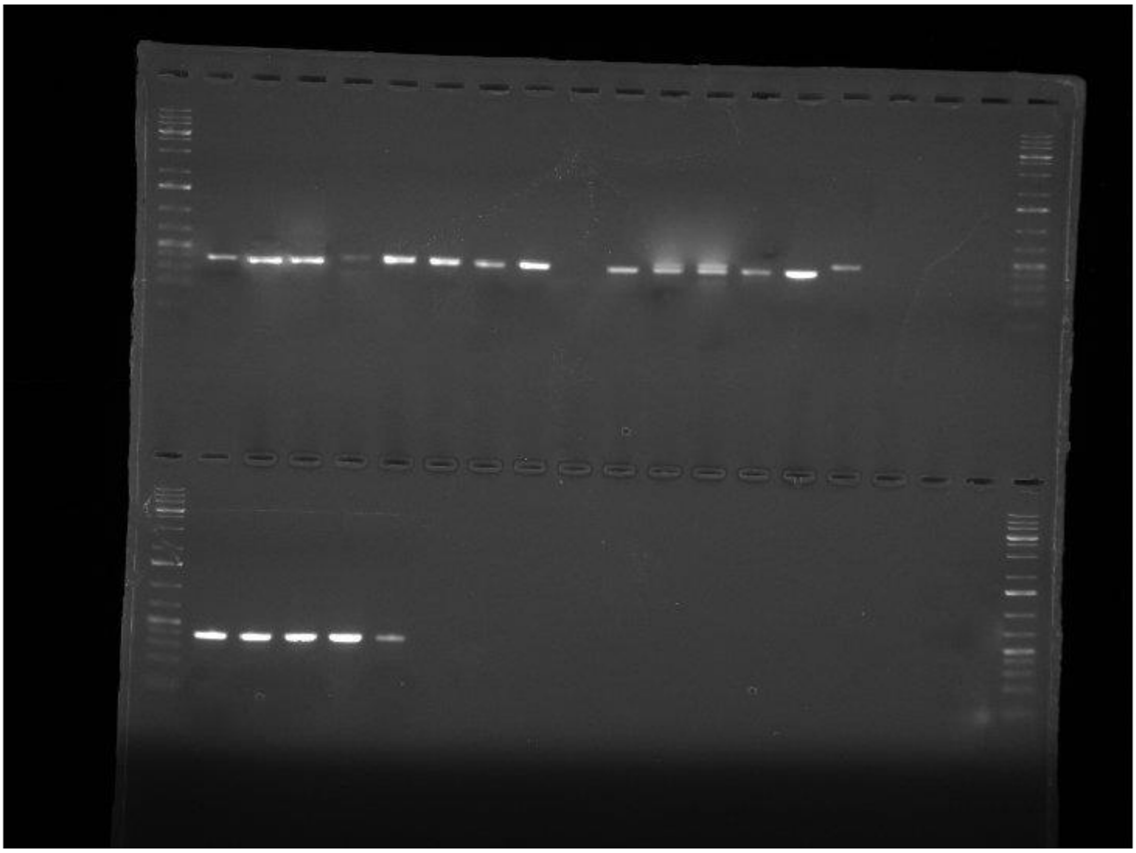

Gel electrophoresis of the *CzcD* gene of bacterial isolates from mining soil. It shows the predicted 398 bp amplicons for positive isolates and negative for the control

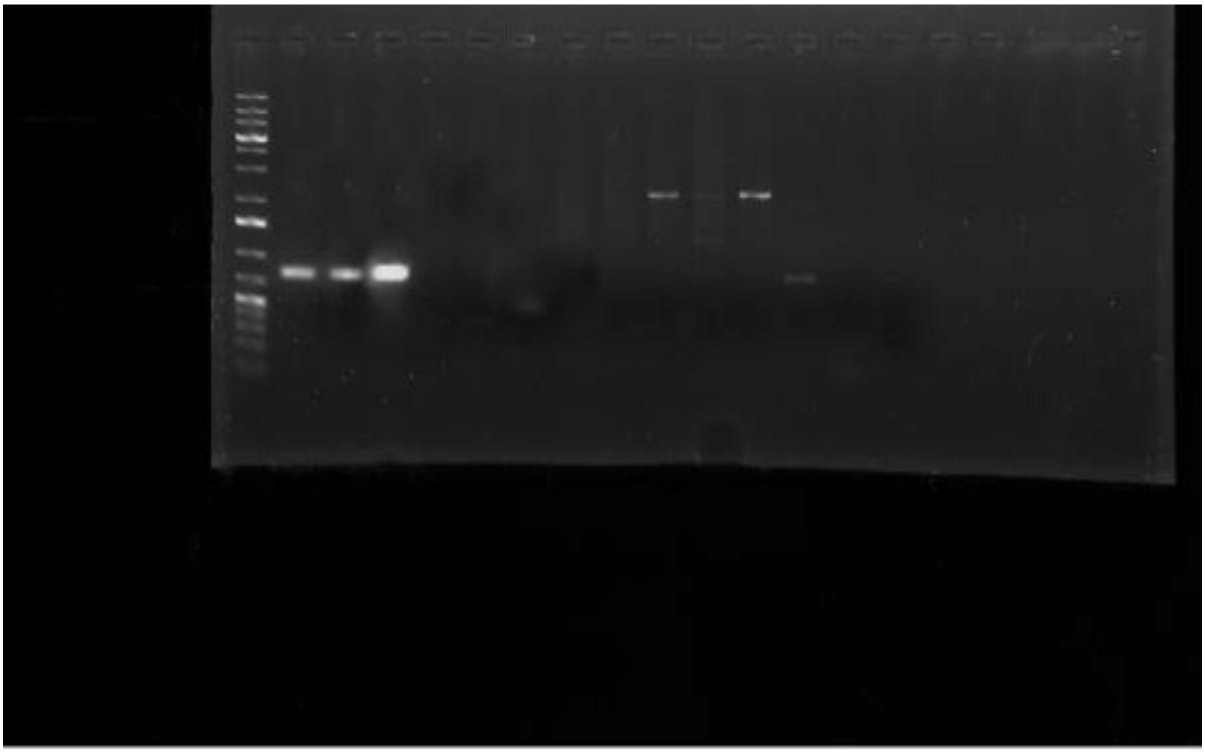

Gel electrophoresis of the *PbrA* gene of bacterial isolates from mining soil. It shows the predicted 756 bp amplicons for positive isolates and negative for the control

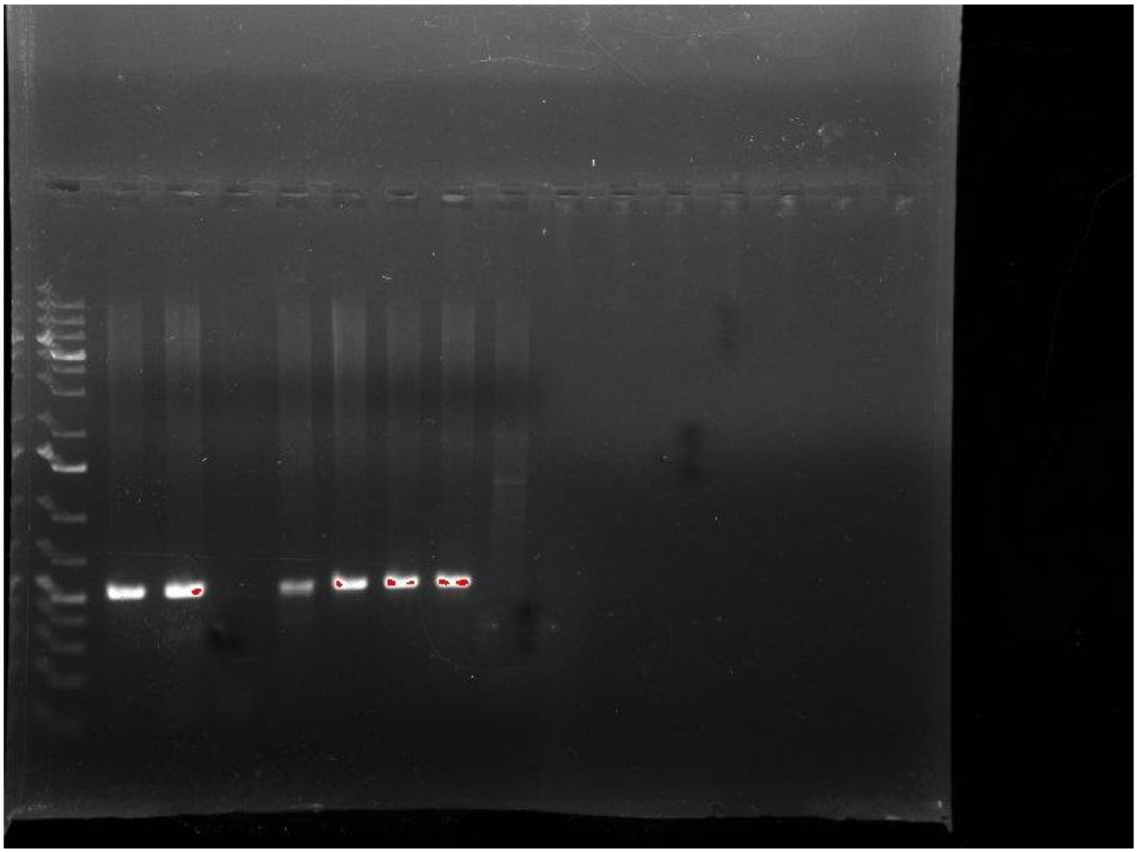

Gel electrophoresis of the *cadA* gene of bacterial isolates from mining soil. It shows the predicted 600 bp amplicons for positive isolates and negative for the control

References

1 Marin, M. *et al.* The role of universal 16S rRNA gene PCR and sequencing in the diagnosis of prosthetic joint infection. *Journal of Clinical Microbiology* **50**, 583-589 (2011).

2 Nies, D. H., Nies, A., Chu, L. & Silver, S. Expression and nucleotide sequence of a plasmid-determined divalent cation efflux system from *Alcaligenes eutrophus*. *Proceedings of the National Academy of Sciences* **86**, 7351-7355 (1989).

3 Abou-Shanab, R., Van Berkum, P. & Angle, J. Heavy metal resistance and genotypic analysis of metal resistance genes in Gram-positive and Gram-negative bacteria present in Ni-rich serpentine soil and in the rhizosphere of *Alyssum murale*. *Chemosphere* **68**, 360-367 (2007).

4 Shin, M.-N. *et al.* Characterization of lead resistant endophytic *Bacillus* sp. MN3-4 and its potential for promoting lead accumulation in metal hyperaccumulator *Alnus firma*. *J. Hazard. Mater.* **199**, 314-320 (2012).

5 Nies, A., Nies, D. H. & Silver, S. Nucleotide sequence and expression of a plasmid-encoded chromate resistance determinant from *Alcaligenes eutrophus*. *J. Biol. Chem.* **265**, 5648-5653 (1990).

6 Dell'Amico, E., Mazzocchi, M., Cavalca, L., Allievi, L. & Andreoni, V. Assessment of bacterial community structure in a long-term copper-polluted ex-vineyard soil. *Microbiol. Res.* **163**, 671-683, doi:https://doi.org/10.1016/j.micres.2006.09.003 (2008).
